# Emerging mechanisms and implications of m6A in CVDs: potential applications of natural products

**DOI:** 10.3389/fcvm.2025.1559064

**Published:** 2025-06-30

**Authors:** Hui Wang, Yun Zhang, Xiao Jiang, Guichun Liu, Shujian Xu, Jinbiao He, Xudong He, Ting Xiao, Lijuan Wang, LiJing Xiao, Xinhui Li

**Affiliations:** ^1^ The Second Affiliated Hospital, Hunan University of Chinese Medicine, Changsha, China; ^2^ The First Clinical Medical College, Yunnan University of Traditional Chinese Medicine, Kunming, China; ^3^ College of Traditional Chinese Medicine, Hunan University of Chinese Medicine, Changsha, China; ^4^ Yunnan Characteristic Plant Extraction Laboratory, School of Chemical Science and Technology, Yunnan University, Kunming, China

**Keywords:** m6A methylation, cardiovascular diseases, natural products, biological processes, epigenetic modification

## Abstract

N6-methyladenosine (m6A) RNA methylation is the most common, abundant, and reversible epigenetic modification of RNAs, and it directs many essential processes in RNAs post-transcriptionally. It has been shown that m6A modification affects cardiovascular diseases (CVDs) by regulating RNA splicing, localization, translation, and stabilization and regulating processes such as autophagy, apoptosis, inflammatory responses, and oxidative stress. A growing body of evidence has shown that natural products have the unique advantage of being highly effective with few side effects and have significant effects against CVDs. However, few studies have explored the relationship between natural products and m6A modification in the development of CVDs. In this review, we summarized the biological functions of m6A modification and discussed the potential mechanisms and processes of m6A modification in various CVDs; we also summarized the research progress of natural products modulating m6A modification in the treatment of CVDs. This review further elucidated the relationship between the modification of m6A methylation and CVDs, thereby contributing to the development of potential therapeutic agents from natural products.

## 1 Introduction

Cardiovascular diseases (CVDs) refer to a series of diseases of the heart and circulation, including hypertension, atherosclerosis, myocardial infarction (MI), pulmonary hypertension (PH), and heart failure (HF), and they have been found as the primary cause of death and rising healthcare costs worldwide (1, 2). According to the 2018 Heart Disease and Stroke Statistical Report released by the American Heart Association, CVDs cause 17.9 million deaths annually, and this figure is expected to exceed 23.8 million by 2030 (3). Although lipid-lowering drugs, antiplatelet drugs, anticoagulation therapy, and percutaneous coronary intervention (PCI) have been widely use in the treatment of CVDs over the past decade, CVDs remain the leading cause of death worldwide, which may be related to the unclear pathogenesis of these diseases. Thus, it is still challenging to reduce the morbidity and mortality related to CVDs effectively.

Epigenetics is a rapidly advancing field of biomedical research. It refers to the heritable changes in gene expression that are not caused by alterations in DNA sequence, including DNA methylation, RNA methylation, chromatin remodeling, and non-coding RNA, and has attracted tremendous research interest owing to its critical role in biological processes such as gene expression, protein function, biological senescence, and disease development (4, 5). To date, more than 170 types of essential modifications of messenger RNA (mRNA) and non-coding RNA (ncRNAs) have been reported, such as N6-methyladenosine (m6A) modification, N1-methyladenosine (m1A) modification, and 5-methylcytosine (m5C) modification (6, 7). Among them, m6A has been one of the most common and abundant epigenetic modifications of eukaryotic mRNA since 1974 (8–10). More than 7,000 mRNAs in mammalian cells are m6A-modified, and it is estimated that m6A exists in 0.1%–0.4% of adenosines (11). Recent studies have found that in addition to mRNAs, m6A also occurs in ribosomal RNAs (rRNAs), transfer RNAs (tRNAs), small nucleolar RNAs, microRNAs (miRNAs), and long non-coding RNAs (lncRNAs) (12, 13).

Most of our knowledge about epigenetics comes from cancer research, but recently, there has been a marked increase in interest in cardiovascular epigenetics (14). Recent studies have revealed that m6A is closely related to the onset and development of CVDs, providing a new insight into the development of intervention strategies for CVDs (15). With the aid of molecular biology technology, we may explore the role of genetics in the pathophysiology of CVDs and develop gene therapies for these diseases. The regulation of m6A methylation may be a promising approach for reversing the progression of CVDs. Therefore, it is necessary to study the relationship between m6A and CVDs, especially the mechanisms involved in m6A modification.

In recent years, natural products have been widely used to prevent and treat a variety of diseases due to their good safety profile and high application value. Natural products represent a large family of diverse chemical entities with a wide range of biological activities; they are mainly obtained from edible and medicinal plants and are widely accepted as complementary and alternative therapies in many countries. It has been shown that natural products are effective in stimulating autophagy, reducing oxidative stress and inflammatory responses, inhibiting apoptosis, and delaying ventricular remodeling and ischemia-refocused injury that accompany CVDs (16). However, there are few reports on natural products that treat CVDs by regulating m6A modification.

To this end, we summarized the biological functions of m6A modification in CVDs. Firstly, we introduced the biological function of m6A modification. Then, we reviewed the mechanisms and processes of m6A modification and discussed its potential role in CVDs. We also summarized the research progress of natural products treating CVDs by modulating m6A modification. This review further elucidated the relationship between the modification of m6A methylation and CVDs, thereby contributing to the development of potential therapeutic agents and intervention strategies, which is of great clinical implication.

## 2 Overview of m6A methylation machinery

**Figure 1:**
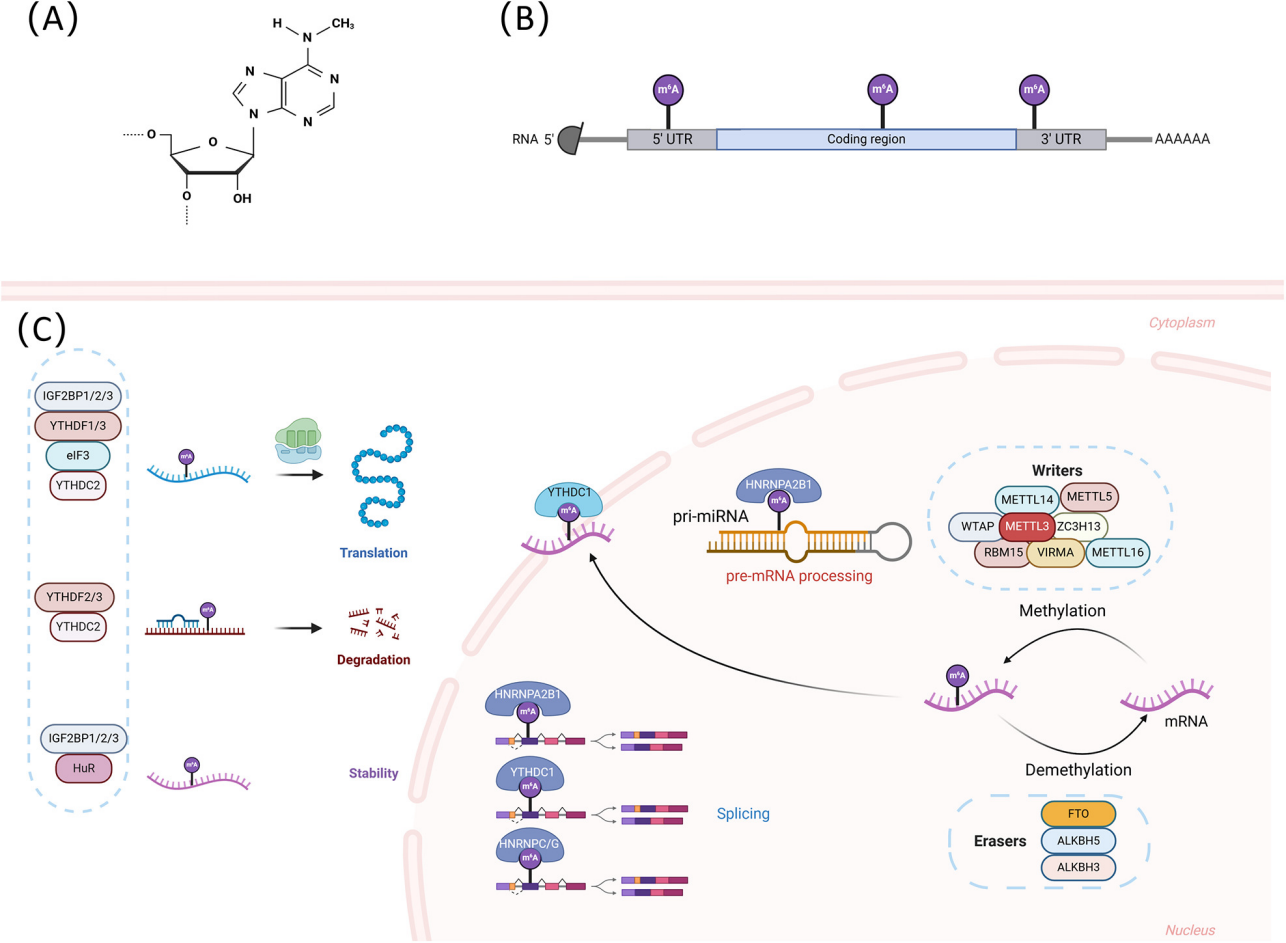
**(A)** The structure of m6A modification. m6A modification refers to the methylation of the sixth N atom of adenine in RNA molecules. **(B)** The regions where m6A modification is mainly enriched. **(C)** The underlying mechanisms of m6A modification. m6A is established by m6A methyltransferases (writers) and removed by m6A demethylases (erasers). m6A readers are involved in various nuclear and cytoplasmic processes in the RNA life cycle, including splicing, export, stability, degradation, and translation through recognizing and binding to the m6A sites of RNAs. This figure was created with BioRender (https://biorender.com/).

m6A has been identified as the most common internal modification of mRNA in most eukaryotic species. Similar to DNA methylation and histone modification, RNA methylation is a dynamic and reversible modification that regulates gene expression. m6A modification is mainly mediated by three types of molecule regulators, i.e., methyltransferases, demethylases, and m6A-binding proteins, which perform catalysis, erasure, and recognition, respectively (17, 18). The m6A modification is mainly enriched around 3'-untranslated regions (3' UTRs) and 5'-untranslated regions (5' UTRs), near stop codons, and within long internal exons at the highly conserved consensus motif RRACH (R = A/G, H = A/C/U) (19). Although m6A was discovered more than 50 years ago, it was not until 2011, when FTO was identified as m6A demethylase, that it sparked enthusiasm in this field, indicating that m6A can be dynamically regulated (20). Since then, the interactions between methyltransferases, demethylases, and m6A-binding proteins, as well as the mechanisms involved in the regulation of RNAs and a range of physiological and pathological processes such as cell differentiation, self-renewal, apoptosis, and the development of cancers, CVDs, and metabolic disorders, have been progressively uncovered (see details in Figure 1).

### 2.1 The writers—m6A methyltransferases

The methyltransferases, also known as “writers”, participate in the composition of the methyltransferase complex (MTC) and catalyze m6A modification. The MTC, consisting of methyltransferase-like3 (METTL3), methyltransferase-like14 (METTL14), Wilms tumor 1 associated protein (WTAP), and other binding partners such as zinc finger CCCH-type containing 13 (ZC3H13), vir like m6A methyltransferase associated (VIRMA), and RNA-binding motif protein 15 (RBM15), recognizes the RRACH sequence in RNAs and catalyzes adenosine modification to m6A (21–25).

The METTL3 protein consists of 580 amino acids and is a critical enzyme in the complex; it contains a zinc finger domain and a methyltransferase domain, both of which are necessary for enzyme activity. METTL3 also has an S-adenosylmethionine binding domain, which transfers methyl from the m6A methylation substrate to the sixth N of adenosine (26). Moreover, METTL3 is the only catalytic subunit in the m6A methyltransferase complex; thus, deletion of this subunit inactivates the complex (27). Although METTL14 shares 43% sequence homology with METTL3, METTL14 has no catalytic activity. METTL14 has an arginine-glycine-glycine structural domain at the C-terminus, which primarily provides an RNA-binding scaffold to improve the stability and catalytic ability of the complex (28). METTL3 and METTL14 form stable heterodimers to regulate the stability of the complex and maintain METTL3 stability and the three-dimensional conformation required for METTL3 enzyme activity (29). WTAP, which has been identified as the third component of the methylation complex, is responsible for the interaction with the METTL3-METTL14 complex. WTAP is essential for initiating and directing the localization of nuclear speckles, which are required for the activation of m6A methylation (30). RBM15 is an interacting partner of WTAP that recruits the m6A writer complex to the u-rich region of mRNAs to repress adapter proteins (31). VIRMA is another important subunit of the writer complex that localizes the m6A modification in the 3'UTR near the termination codon (32). ZC3H13 regulates nuclear m6A methylation through its association with WTAP and RBM15 (33).

New methylation-related enzymes have also been discovered recently. METTL5 binds to the methyltransferase activator TRMT112 to form a heterodimer and is involved in m6A modification of 18S ribosomal RNA (34), and METTL16 regulates the activity of all cellular methyltransferases, including METTL3/METTL14 (35). RBM15 and RBM15B, which are other components of the mRNA methylation machinery, are RNA-binding proteins that bind specifically to the region of mRNA and regulate the addition of m6A to the DRACH consensus sequence (36). Current studies on m6A methyltransferases are still in the exploratory stage, and more components and functions will be discovered as research progresses.

### 2.2 The erasers—m6A demethylase

Demethylation refers to the removal of m6A modification from RNA molecules by demethylating the sixth nitrogen atom of adenylate, which is a crucial step in the reversal of m6A modification and reveals that m6A methylation is a dynamic and reversible process (6). Three m6A demethylases have been identified in eukaryotes, namely fat mass and obesity-associated (FTO), alpha-ketoglutarate-dependent dioxygenase alkB homolog 5 (ALKBH5), and alpha-ketoglutarate-dependent dioxygenase alkB homolog 3 (ALKBH3); all of them are located in the nucleus, and their knockdown can significantly increase the level of m6A methylation (37).

As a potent regulator of nuclear mRNA processing events, FTO binds preferentially to pre-mRNAs in intronic regions, in close proximity to selectively spliced exons and poly(A) sites, and is involved in selective splicing and 3'end mRNA processing (38). The function of FTO differs depending on whether it is in the nucleus or the cytoplasm. The primary function of FTOs in the nucleus is m6A modification within mRNAs, whereas FTOs in the cytoplasm also mediate demethylation of m6A modification within mRNAs (39). FTO-dependent demethylation of m6A regulates mRNA stability, degradation, and translational efficiency, thereby contributing to the changes in protein levels, which is essential for the development of the cardiovascular system (40). The AlkB family is characterized by ferrous- and 2-oxoglutarate-dependent nucleic acid oxygenases. Similar to FTO, ALKBH5 is also a potent m6A demethylase and co-localizes in nuclear speckles with other mRNA processing factors (41). When ALKBH5 is knocked down, the level of mRNA m6A modification increases in the nucleus, while the amount of mRNA decreases in the cytoplasm, suggesting that ALKBH5 may be involved in mRNA transport (37). ALKBH3 has been found to be involved in a similar process (42). Both of them play a critical role in biological processes such as cell cycle, stress response, and apoptosis.

### 2.3 The readers—m6A RNA-binding proteins

m6A RNA-binding proteins (RBPs) specifically recognize target m6A-modified mRNAs and subsequently play a role in mRNA splicing, folding, transport, translocation, degradation, translation, and stability; they are also involved in the regulation of RNA metabolism and biological processes (43). The YTH family, the best-known m6A RNA-binding protein, includes the YTH domain-containing family protein 1–3 (YTHDF1/2/3) and YTH domain-containing protein 1–2 (YTHDC1/2) subfamilies. YTHDF1 translocates from the cytoplasm to the nucleus, initiates and enhances translation in a eukaryotic initiation factor 3 (eIF3)-dependent manner, and promotes cap-independent translational regulation of m6A-modified RNA transcripts in the 5'UTR region, thereby increasing translational efficiency and facilitating protein synthesis (44). YTHDF2 recruits methylated transcripts to mRNA decay sites, which are processed with processing body markers to promote their degradation. Finally, YTHDF3 synergizes with YTHDF1 and YTHDF2 to accelerate m6A-mediated translation or degradation (45). YTHDC1 regulates RNA nuclear export (46) and splicing (47), while YTHDC2 modulates the translation and abundance of target genes (48).

In addition, the structural domains of the heterologous nuclear ribonucleoprotein (HNRNP) and insulin-like growth factor 2 mRNA binding proteins (IGF2BPs) are also essential components of recognition proteins. The HNRNP family, which includes HNRNPA2B1, HNRNPC, and HNRNPG, does not directly recognize m6A but promotes pre-mRNA processing by selectively binding to m6A-modified transcripts. Among them, HNRNPA2B1 is a nuclear m6A reader that binds directly to nuclear transcripts, eliciting regulatory effects on RNA splicing and promoting primary miRNA processing; HNRNPG interacts with RNA polymerase II and m6A-modified pre-mRNA to regulate selective splicing and the expression of target mRNA (49, 50). IGF2BPs include IGF2BP1, IGF2BP2, and IGF2BP3. Functionally, IGF2BPs recognize m6A, preferentially bind to UGGAC consensus sequences containing the m6A core motif GGAC, and increase the stability and the translation efficiency of target m6A-modified mRNAs (51). HuR is an RNA-binding protein that recognizes U/AU-rich elements in different RNAs through two RNA recognition motifs, RRM1 and RRM2. HuR tightly regulates the fate of target RNA at the post-transcriptional level and binds to the m6A sites of ncRNAs to stabilize their transcripts (52). Understanding the specific and redundant functions and the targets of RBPs may help to identify and regulate more m6A methylation-mediated functions in cellular homeostasis. Interestingly, ribosomes may also function as m6A readers. It has been shown that bacterial ribosomes stall on mRNAs containing m6A codons, while ribosome mapping data suggest that mammalian ribosomes also tend to stall on m6A sites (53).

### 2.4 Methods to detect m6A

The development of sequencing technologies has facilitated epigenetic studies and helped us better assess the methylation status of m6A sites. Dot blotting and colorimetric methods are commonly used to observe the overall changes of m6A at the level of the whole transcriptome, with the advantage of being able to achieve quantitative or semi-quantitative estimates; however, these approaches cannot locate m6A sites accurately (54, 55). With the use of methylated RNA immunoprecipitation and next-generation sequencing (MeRIP-Seq), mRNAs were extracted from the cell, broken into ∼100nt fragments, immunoprecipitated using antibodies to mA, attached to sequencing junctions, reversely transcribed into cDNA, and amplified using PCR; this is followed by high-throughput sequencing. However, this approach limits the resolution of the m6A modification site and precludes single-base detection (18). Therefore, in 2015, Jaffrey et al. developed a method based on m6A antibody crosslinking combined with immunoprecipitation to determine m6A sites with single-base resolution. The method makes use of a combination of m6A and the corresponding antibody, and then induces crosslinking between the antibody and RNA using UV light, which leads to changes in base-pairing or the termination of reverse transcription; this results in a mutation or truncation of cDNA, thereby achieving single-base resolution. However, this method is highly antibody-dependent (56). The glyoxal and nitrite-mediated deamination of unmethylated adenosine (GLORI) technology, which was developed by Wang Jing et al. in 2023, does not rely on antibodies but rather on a combination of chemical screening. Using this approach, Wang et al. found that the unmethylated adenosine was efficiently deamidated to form inosine by the catalytic system of glycolaldehyde and that after sequencing, m6A was still read as A, achieving single-base identification of m6A. GLORI detects m6A absolutely and quantitatively without preference for a single base due to its characteristics of high sensitivity and high specificity, which breaks through the bottleneck of the current quantitative sequencing technology (57). In addition, a variety of novel detection methods are emerging, and nanomaterials such as quantum dots and nanocluster beacons have been used in studies of methylation detection owing to their unique properties (58, 59).

The above research advances have provided technical support for the localization of m6A modification and revealed dynamic mRNA modification on gene expression. However, there are still difficulties and challenges in single nucleotide detection and accurate quantitative sequencing in the cardiovascular field, which necessitates further research.

## 3 Regulatory mechanisms of m6A methylation

**Figure 2:**
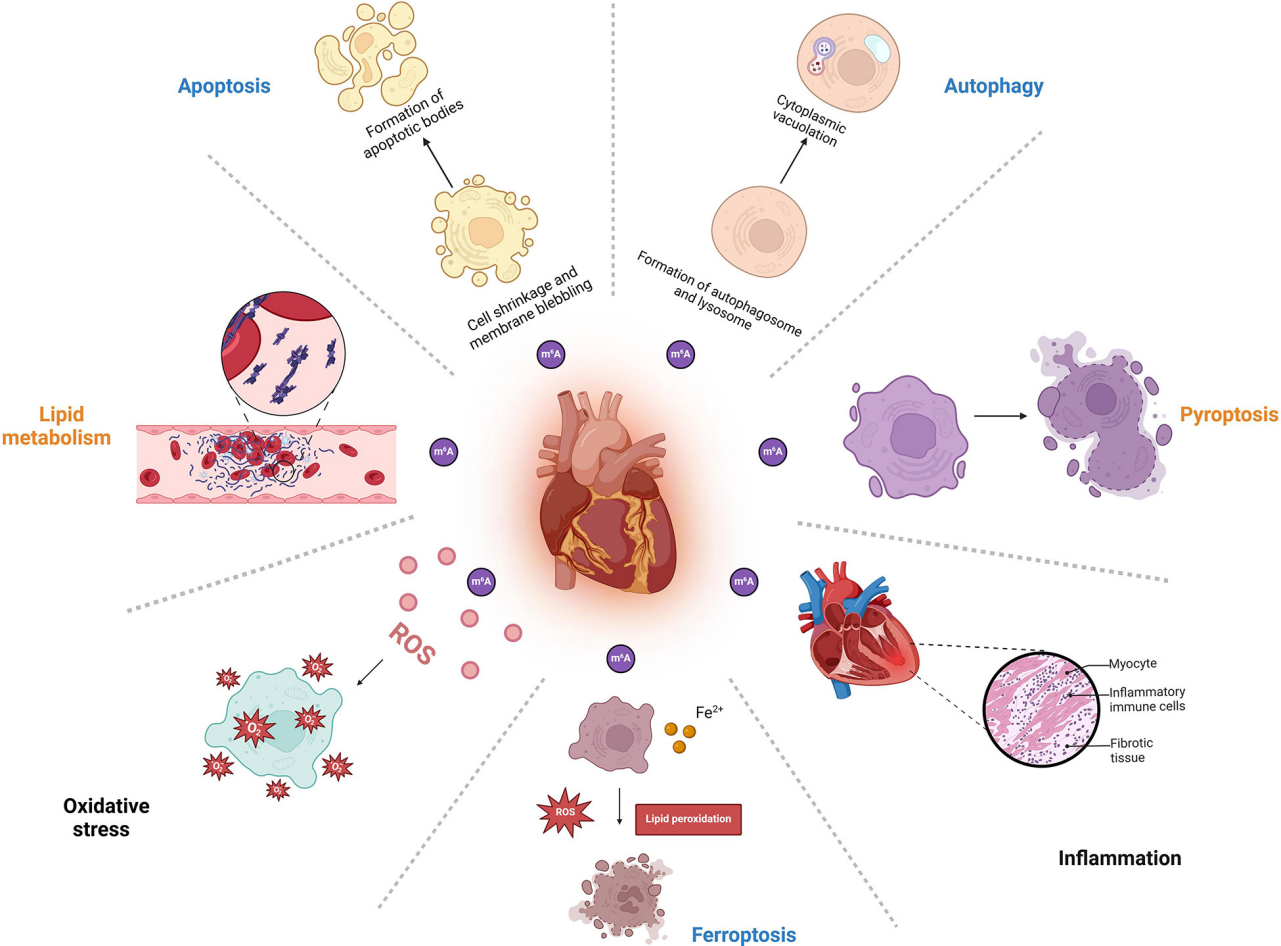
The mechanisms of m6A modification in cardiovascular diseases. This figure was created with BioRender (https://biorender.com/).

m6A methylation is involved in the regulation of different biological processes in an enzyme activity-dependent manner. It plays essential roles in myocardial development, energy metabolism, stress response, and the maintenance of cardiac homeostasis. Given the wide-ranging role of m6A in maintaining homeostasis, its dysregulation may result in disorders such as cardiovascular diseases. In the next section, we will focus on the associations of m6A with apoptosis, autophagy, pyroptosis, oxidative stress, inflammatory response, lipid metabolism, and ferroptosis, which may provide new insights into the pathogenesis and management of CVDs (see details in Figure 2 and Table 1).

**Table 1:** The regulatory mechanisms of m6A methylation.

Cellular process	m6A regulator(s)	Function	Mechanism(s)	Application(s)	Reference
Apoptosis↑	METTL3↑	Writer	Increases the level of m6A methylation of TFEB mRNA, accelerates the degradation of the TFEB mRNA and inhibits TFEB expression	Ischemia-reperfusion	(60)
Apoptosis ↓	FTO ↑	Eraser	Causes an upregulation of MHRT and reduced m6A modification of MHRT	Heart failure	(61)
Apoptosis↑	ALKBH5 ↑	Eraser	Activates post-transcriptional FOXO3	Diabetic cardiomyopathy	(62)
Autophagy ↓	METTL3 ↑	Writer	Reduces autophagic fux by regulating TFEB levels and transcriptional activity,	Ischemia-reperfusion	(60)
Autophagy ↑	ALKBH5 ↑	Eraser	Increases the levels of TFEB pre mRNA	Ischemia-reperfusion	(60)
Pyroptosis↓	METTL3↓	Writer	Increases the expression of hsa_circ_0029589	Acute coronary syndrome	(70)
Pyroptosis↓	METTL14↓	Writer	Binds to the m6A site of NEAT1 and decrease NEAT1 expression	Atherosclerosis	(71)
Pyroptosis↓	METTL14 ↑	Writer	Inhibits the expression of TINCR and the stability of NLRP3	Diabetic cardiomyopathy	(72)
Inflammatory responses↓	METTL3↑	Writer	Inhibits TNF-α-mediated phosphorylation of p65 in endothelial cells, inhibit the NF-κB inflammatory pathway	Atherosclerosis	(73)
Inflammatory responses↑	METTL14↑	Writer	Through NF-κB/IL-6 signaling	Atherosclerosis	(76)
Inflammatory responses↑	METTL14↑	Writer	Increases m6A modification on FOXO1, thereby increasing adhesion molecule expression,	Atherosclerosis	(77)
Inflammatory responses↑	FTO↓	Eraser	Increases the methylation of mRNA transcripts of inflammatory cytokines such as IL-6, TNF-α, IL-1β	Myocardial inflammation	(80)
Inflammatory responses↓	FTO ↓	Eraser	Decreases the expression of STAT1 in M1-polarised macrophages and the expression of STAT6 and PPAR-γ in M2-polarised macrophages.	Macrophage M1 and M2 polarisation	(83)
Inflammatory responses↑	YTHDF2↑	Reader	Degrades the Hmox1 mRNA	Pulmonary hypertension	(84)
Oxidative stress↑	METTL3↑	Writer	Increases the stability and expression level of lncRNA ABHD11-AS1	Ischemia-reperfusion injury	(85)
Oxidative stress↓	ALKBH5↑	Eraser	Suppresses m6A methylation of MG53 and inhibited MG53 degradation	Myocardial infarction	(87)
Lipid metabolism↑	FTO↑	Eraser	Acts in the 3′UTR region of mRNA of several adipogenic genes	Hyperlipidemia	(89)
Lipid metabolism↓	FTO ↓	Eraser	Increases the expression of prostaglandin D synthase	Hypertension	(91)
Lipid metabolism↓	METTL3↑	Writer	Increases in PPARα mRNA level	Lipid metabolism disorders	(92)
Lipid metabolism↑	METTL14↓	Writer	Inhibits the expression of SR-B1 and decrease the m6A methylation of SR-B1 mRNA	Lipid metabolism disorders	(93)
Ferroptosis↓	METTL14↓	Writer	Reduces the stability of KCNQ1OT1	Doxorubicin-induced cardiotoxicity	(97)
Ferroptosis↑	FTO ↓	Eraser	Upregulates BACH1 expression and modificate BACH1 m6A	Septic cardiomyopathy	(99)
Ferroptosis↓	FTO ↑	Eraser	Activates P21/Nrf2 in a P53-dependent or -independent manner	Doxorubicin-induced cardiotoxicity	(100)
Ferroptosis↓	ALKBH5 ↑	Eraser	Decreased ACSL4 levels	Atherosclerosis	(101)

### 3.1 Regulation of apoptosis

Apoptosis is an intrinsically inherited form of programmed cell death that occurs as a morphological alteration of cell sequestration and is closely related to atherosclerosis and ischemic cardiomyopathy. Numerous studies have confirmed the role of m6A methylation as a biomarker for apoptosis. It has been proposed that m6A methylation regulates apoptosis through the modulation of apoptosis-related genes, silencing of methylated or demethylated enzyme genes, and the mediation of transcripts. Studies have shown that ischemia/reperfusion (I/R) increases the level of m6A methylation of the transcription factor EB (TFEB) mRNA by inducing the expression of METTL3; this accelerates the degradation of the TFEB mRNA, inhibits TFEB expression, impairs autophagic flux, and promotes apoptosis, while silencing METTL3 enhances autophagic flux and inhibits apoptosis in H/R-treated cardiomyocytes (60). A study found that FTO overexpression led to up-regulation of myosin heavy chain-associated RNA (MHTR) and reduced m6A modification of MHTR in H/R-treated myocardial cells, that FTO up-regulation repressed apoptosis of H/R-treated myocardial cells, and the knockdown of FTO could lead to the opposite results (61). Shao et al. (62) found that the level of ALKBH5 was up-regulated in cardiac tissues of mice with diabetic cardiomyopathy and activated post-transcriptional forkhead box O3 (FOXO3) in an m6A-YTHDF2-dependent manner, leading to an increased level of cardiomyocyte apoptosis. Point mutations targeting the m6A modification site of the target transcript can also regulate apoptosis using gene editing techniques (63). Despite the current advances in understanding the role of m6A methylation, there are still limitations in studies on its regulation of apoptosis, and the mechanism of interaction between downstream targets remains to be further investigated.

### 3.2 Regulation of autophagy

Autophagy is also believed to be closely associated with CVDs. Several studies have found that m6A modification affects the transcriptional regulation of autophagy-related genes, playing a role in autophagosome formation and regulation (64). In 2018, it was first reported that FTO regulated autophagy by affecting the abundance of Unc-51, such as Unc-51-like autophagy-activated kinase 1 (ULK1) (65). The overexpression of FTO reduces the translation of mRNAs through m6A demethylation, inhibits the activation of the mammalian target of the rapamycin (mTOR) signaling pathway, and up-regulates the level of ULK1 protein, which in turn facilitates the initiation of cell autophagy (66). The knockdown of FTO, on the other hand, causes decreased expression of the key protein light chain 3B II (LC3BII) in the autophagy process, and increased level of the autophagy substrate P62 (65). m6A acts as an inhibitor that enhances METTL3 and reduces autophagic flux by regulating TFEB levels and transcriptional activity, whereas ALKBH5 plays an opposite role in Hypoxia/reoxygenation (H/R)-treated cardiomyocytes (60). ALKBH5 has been found to enhance autophagy by reducing m6A methylation in FIP200 transcripts (67). Notably, the regulation of autophagy by m6A methylation is complex and dynamic, as well as tissue- and disease-specific. For example, m6A methylation in CVDs not only suppresses ischemia/hypoxia-induced cellular autophagy but also promotes autophagy by accelerating its initiation. Therefore, further investigation the relationship between m6A modification and autophagy in cardiovascular processes may provide new therapeutic approaches for CVDs.

### 3.3 Regulation of pyroptosis

Pyroptosis is a form of programmed cell death characterized by rapid plasma membrane disruption and the subsequent release of cellular contents and proinflammatory mediators (68). It has been found that phosphatase and tensin homolog (PTEN) mRNA containing the m6A locus is involved in the cryo-activated phosphatidylinositol 3-kinase (PI3K)/protein kinase B (Akt)/glycogen synthase kinase-3 (GSK-3β) signaling pathway, which down-regulates the expression of pyroptosis-related proteins, such as NOD-like receptor protein 3 (NLRP3) and apoptotic speck-like protein containing CARD (ASC), and further protects hippocampal neurons from hypoxia/reoxygenation-induced pyroptosis (69). A study of Guo et al. (70) found that the relative RNA expression level of hsa_circ_0029589 was reduced, whereas the m6A level of hsa_circ_0029589 and the expression of METTL3 were significantly elevated in macrophages of patients with acute coronary syndrome (ACS). This study also found that overexpression of IFN regulatory factor-1 (IRF-1) suppressed the expression of hsa_circ_0029589 in macrophages but induced the expression of m6A and METTL3. Moreover, both overexpression of hsa_circ_0029589 and inhibition of METTL3 significantly attenuated macrophage pyroptosis, indicating the key role of m6A modification in macrophage pyroptosis. Although the study is still in its infancy, this newly discovered regulatory network may provide promising therapeutic options for ACS. A recent study found that exercise-induced significant down-regulation of m6A modification and METTL14, which bind to the m6A site of nuclear paraspeckle assembly transcript 1 (NEAT1) and decrease NEAT1 expression, thereby mitigating endothelial cell pyroptosis and atherosclerosis (71). Interestingly, another study revealed the opposite relationship between METTL14 and pyroptosis in CVDs. Meng et al. (72) found that METTL14 inhibited pyroptosis and diabetic cardiomyopathy by mediating the m6A modification of terminal differentiation-induced non-coding RNA (TINCR), which inhibits the expression of TINCR and the stability of NLRP3. These results suggest that m6A is actively involved in pyroptosis and may assess the prognosis of CVDs. However, m6A-modifying enzymes are a double-edged sword in regulating pyroptosis, which may depend on the abundance of their RNA-binding proteins as well as their recognition sites. Thus, it remains possible that m6a-mediated focal death may have other defects yet to be discovered.

### 3.4 Regulation of inflammation response

Inflammation response leads to vascular endothelial damage and thrombosis, which may underlie the mechanisms of CVDs. Chien et al. (73) found that METTL3 could effectively inhibit TNF-α-mediated phosphorylation of p65 in endothelial cells, inhibit the nuclear transcription factor κB (NF-κB) inflammatory pathway to reduce the inflammatory response of endothelial cells, and effectively inhibit the adhesion of monocytes to endothelial cells, thereby suppressing the development of atherosclerosis. Similarly, it was found that lipopolysaccharide (LPS) stimulation promotes the expression and bioactivity of METTL3 in macrophages, and the overexpression of METTL3 alleviates LPS-induced inflammation through the NF-κB signaling pathway, further demonstrating the relationship between m6A methylation and inflammation (74). Overexpression of ALKBH5 can increase the expression of TNF receptor family member 9, a trigger factor of the NF-κB pathway, and promote the expression of NF-κB, thereby enhancing inflammation response (75). METTL14 promotes inflammatory responses in atherosclerosis-associated macrophages through NF-κB/IL-6 signaling pathway (76) and also increases m6A modification of forkhead box O1 (FOXO1), thereby increasing adhesion molecule expression, mediating endothelialmonocyte adhesione (77). Overexpression of FTO has been found to inhibit the expression of NF-κB through related pathways (78) and reduce the expression of inflammatory vesicle NLRP3 (79), thus reducing the level of inflammation. Similar to the results of this study, reduced expression of FTO has also been found in myocardial tissues of mice with myocardial inflammation, and FTO knockdown can lead to hypermethylation of mRNA transcripts of inflammatory cytokines, resulting in an increase in the levels of inflammatory factors such as interleukin-6 (IL-6), tumor necrosis factor α (TNF-α), and interleukin-β (IL-1β) (80). The latest research has also revealed that YTHDF2 is tightly associated with macrophage polarization in inflammation (81, 82). According to a recent study, FTO silencing significantly inhibited macrophage M1 and M2 polarization and FTO knockdown resulted in down-regulated expression of signal transducer and activator of transcription1 (STAT1) in M1-polarised macrophages, whereas the expression of STAT6 and peroxisome proliferator-activated receptor gamma (PPAR-γ) was decreased in M2-polarised macrophages. Furthermore, the stability and expression of STAT1 and PPAR-γ mRNA also increased when YTHDF2 was silenced. FTO knockdown inhibits the NF-κB signaling pathway and reduces the stability of STAT1 and PPAR-γ mRNA through the involvement of YTHDF2, thereby hindering macrophage activation (83). m6A modification and YTHDF2 recognition were found on heme oxygenase 1 (Hmox1) mRNA in alveolar macrophages during the development of pulmonary hypertension. YTHDF2 can contribute to pulmonary hypertension through the degradation of Hmox1 mRNA, early macrophage polarization, and vascular inflammation (84). These results suggest that m6A may play an essential role in macrophage activation. Given the critical role of macrophage polarization in the pathogenesis of various inflammatory cardiovascular diseases, it is significant to further explore the contribution of m6A methylation to the progression of cardiovascular diseases.

### 3.5 Regulation of oxidative stress

Oxidative stress refers to an imbalance in the oxidative or antioxidant system of the organism and is associated with excessive production of reactive oxygen species (ROS) in organisms. High level of ROS can result in cellular structural changes and genetic damage, which are closely associated with cardiomyocyte death and subsequent changes in the structure and function of the heart. METTL3 m6A-dependently increased the stability and expression level of lncRNA ABHD11-AS1, which further upregulated hypoxia inducible factor 1 alpha inhibit (HIF1AN) by sponging miR-1301-3p and facilitated the development of oxidative stress and cerebral I/R injury (85). METTL3 and METTL14 synergistically interact with other forms of mRNA methylation (e.g., m5C) to increase the translational level of p21, thus affecting its expression during oxidative stress and promoting oxidative stress-induced cellular senescence (86). A recent study found that ALKBH5 expression was down-regulated in patients with AMI, I/R-induced mice, and H/R-induced cardiomyocytes. Overexpressed ALKBH5 bound to mitsugumin (MG53), inhibited m6A methylation and MG53 degradation, increased mRNA stability, suppressed H/R-induced oxidative stress, and inhibited I/R-induced collagen deposition, myocardial infarction, and cardiac function (87). As a pathological inducer, low fluid shear stress (FSS) mediates endothelial dysfunction and the development of almost all CVDs. In addition, it has been shown that low FSS may regulate the aging process by altering the m6A modification of mammalian target of rapamycin (mTOR), PI3K/Akt, insulin, and Estrogen Receptor Related Beta (ERRβ) signaling pathways and by hypomethylating the key transcriptional factors in response to oxidative stress, such as HIF1α, nuclear factor of activated T cells 5 (NFAT5), and nuclear factor erythroid 2-related factor 2 (NFE2L2) (88). These results suggest that m6A is actively involved in oxidative stress and may serve as an indicator for disease outcomes.

### 3.6 Regulation of lipid metabolism

Disturbances in lipid metabolism, which lead to excessive lipid accumulation or ectopic tissue lipid accumulation, are common drivers of atherosclerosis. FTO-mediated RNA demethylation can regulate the process of lipid synthesis, acting on the 3'UTR of mRNA of adipogenic genes, thereby affecting the synthesis of triglycerides and cholesterols (89). Overexpression of FTO results in a body- and fat mass-dependent increase leading to obesity, whereas mice deficient in FTO show postnatal growth retardation and reduced food intake with a concomitant reduction in adipose tissue (90). The absence of FTO can prevent high-fat diet-induced insulin resistance and hyperinsulinemia by enhancing AKT phosphorylation and also prevent obesity-induced hypertension by increasing the expression of prostaglandin D synthase, which preserves myogenic tone in resistance arteries (91). In obese mice, m6A methylation and METTL3 expression were reduced, but after elevating METTL3 level led to an increase in PPAR*α* mRNA level which alleviated lipid metabolism disorders (92). Park et al. (93) found that down-regulation of METTL14 could inhibit the expression of the scavenger receptor B-type 1 (SR-B1) through down-regulation of the m6A methylation of SR-B1 mRNA, which further reduced cholesterol efflux, promoted foam cell formation, and aggravated lipid metabolism disorders. Despite the increased recognition of the biological significance of m6A modification, the global effects of m6A regulators on the transcription and translation of lipid metabolism-related genes, as well as the molecular determinants conferring RNA specificity, remain to be better understood.

### 3.7 Regulation of ferroptosis

Ferroptosis is a regulated form of cell death characterized by iron-dependent overaccumulation of lipid hydroperoxides (94). In recent years, ferroptosis has been found to be involved in the development and progression of CVDs (95). Studies have shown that ferroptosis inducers can increase the level of m6A methylation (96), and m6A methylases in turn can also regulate the level of ferroptosis. Zhuang et al. (97) found that overexpressed METTL14 could catalyze the m6A level of KCNQ1 opposite strand/antisense transcript 1 (KCNQ1OT1) and participate in the ferroptosis process of cardiomyocytes, and that silencing METTL14 could reduce the stability of KCNQ1OT1 and suppress the ferroptosis of cardiomyocytes in doxorubicin-induced mice. In addition, related studies found that the m6A-binding proteins, YTHDF1 and YTHDC1, might be endogenous inducers of ferroptosis (98). The relationship between FTO and ferroptosis has also been demonstrated in several studies. For instance, FTO was found to be associated with doxorubicin-induced cardiotoxicity, and FTO overexpression was found to significantly improve cardiac function and cell viability in doxorubicin-treated mice. Activation of ferroptosis in cardiomyocytes has been found in a mouse model of septic cardiomyopathy. Inhibition of FTO expression was found to induce the up-regulation of the expression of BTB domain and CNC homolog 1 (BACH1) and the modification of BACH1 m6A in septic cardiomyopathy, leading to accumulation of iron in mitochondria and overproduction of ROS, which ultimately triggered lipid peroxidation and ferroptosis in cardiomyocytes. In contrast, overexpression of FTO was found to reverse this process, inhibiting ferroptosis and attenuating cardiac inflammation and dysfunction (99). Furthermore, a study showed that FTO inhibited doxorubicin-induced ferroptosis through P21/Nrf2 activation in a P53-dependent or -independent manner by mediating m6A demethylation (100). A recent study also demonstrated that methylation affected ferroptosis by modulating mRNA stability. In human microvascular endothelial cell line-1 (HMEC-1), ALKBH5 overexpression reversed ox-LDL-induced ferroptosis, including the up-regulation of Fe^2+^, lipid ROS, and acyl-CoA synthetase long-chain family member 4 (ACSL4), and the down-regulation of Glutathione (GSH) and Glutathione Peroxidase 4 (GPX4). This suggests that up-regulation of ALKBH5 inhibits ox-LDL-induced ferroptosis in HMEC-1 cells by reducing the mRNA stability of ACSL4 in a demethylation-dependent pathway (101), which may provide a novel therapeutic strategy for the treatment of atherosclerosis. However, ferroptosis, as a novel mode of programmed cell death, has yet to be fully explored. Thus, the role of ferroptosis based on m6A regulation in cardiovascular pathogenesis still needs to be further investigated.

## 4 The role of m6A methylation in cardiovascular diseases

m6A, the most prevalent RNA modification, has been shown to exert its function in a variety of ways, including splicing, export, decay, and translation initiation, to regulate RNA fate. RNA modifications have been shown to be heavily involved in heart development and progression of diseases. Notably, m6A also affects biological processes by upsetting stable base pairing, which controls a number of RNA functions. In this section, we will focus on the role of m6A methylation in CVDs including essential hypertension, atherosclerosis, ischemia/reperfusion injury, and other related conditions, as well as the related pathogenesis (See details in Table 2).

**Table 2:** The role of m6A modification in various cardiovascular diseases.

Disease	m6A regulator(s)	Function	Molecular mechanism(s)	Reference
Essential hypertension	FTO	Eraser	Regulates arterial myogenic contraction and vascular resistance	(91)
Pulmonary hypertension	METTL3	Writer	Affects the m6A level of PH-related genes such as Nog, Vangl2, Trps1	(105)
	FTO, YTHDF1	Eraser and Reader	Regulates inflammation, glycolysis, TGF-βfamily receptor members, ECM receptor interactions, and PDGF signalling pathway	(106)
	METTL3, METTL14	Writer	Inhibits the proliferation and migration of PASMCs	(107)
	YTHDF2	Reader	Promotes PTEN degradation, which in turn activates the PI3K/Akt signaling pathway	(108)
	-	-	m6A methylation affects the progression of PH through circXpo6 and circTmtc3 in the circRNA-miRNA-mRNA network	(109)
Atherosclerosis	METTL3	Writer	Involves in the inflammatory cascade response in ECs	(73)
	METTL3	Writer	Affects protein synthesis and energy metabolism	(112)
	METTL3	Writer	Inhibits the development of endothelial atherosclerosis in the JAK2/STAT3 pathway	(113)
	METTL3	Writer	Promotes m6A-dependent degradation of EGFR mRNA	(114)
	METTL3	Writer	Enhances LncRNA H19 stability	(115)
	METTL14	Writer	Significantly inhibits ECs and atherosclerotic plaque formation	(77)
	METTL14	Writer	Increases the processing of m6A methylation levels in pri-miR-19a, and promoted the proliferation and invasion of vascular ECs	(110)
	METTL14	Writer	Affects the m6A methylation of LncRNA ZFAS1, which is involved in cholesterol metabolism and vascular inflammation,	(116)
Ischemia/reperfusion Injury	METTL3	Writer	Mediates DGCR8 to promote the expression of miR-143-3p and reduce the expression level of protein kinase C epsilon	(117)
	METTL3	Writer	Promotes the localization of eIF4A2 to target genes and facilitates the translation of these genes in hypoxic myocardium	(118)
	WTAP	Reader	Promotes endoplasmic reticulum stress and apoptosis, activated mRNA m6A levels of ATF4, and up-regulated its expression	(119)
	METTL14	Writer	Regulates Wnt1/beta-Catenin Signaling Pathway	(120)
Myocardial infarction	ALKBH5	Eraser	Reduces the level of LDH and CK-MB and improves systolic and diastolic function	(121)
	FTO	Eraser	Improves cardiac homeostasis	(122)
	METTL3	Writer	Decreases m6A-mediated maturation of miR-143-3p, which increased the level of miR-143-3p targets	(123)
Cardiac hypertrophy	METTL3	Writer	Leads to compensatory cardiac hypertrophy	(125)
	YTHDF2	Reader	Degrades the cardiac hypertrophy marker MYH7 mRNA	(126)
	IGF2BP2	Reader	Forms a complex with AGO2 to facilitate miR-133a's accumulation	(127)
Heart failure	FTO	Eraser	Attenuates ischemia-induced cardiac dysfunction in HF mice	(122)
	FTO	Eraser	Modulates glycolysis and glucose uptake	(129)
	FTO	Eraser	Cardiomyocyte-specific knockout of FTO accelerated the progression of HF	(130)

### 4.1 m6A in essential hypertension

Essential hypertension is a significant risk factor for CVDs, and sustained high blood pressure has serious adverse effects on target organs such as the heart and kidneys. It appears to have a higher incidence and prevalence in certain races and families, suggesting a genetic link to the condition. As a result of the interaction between genetic and environmental factors, the pathogenesis of hypertension is also influenced by m6A methylation (102). Kruger et al. (91) found that FTO-mediated m6A played a vital role in regulating arterial myogenic contraction and vascular resistance and that knockdown of FTO genes could attenuate obesity-induced vascular resistance and blood pressure elevation. Using high-throughput sequencing technology, Wu et al. (103) revealed that the abundance of m6A was reduced in microvascular perivascular cells of spontaneously hypertensive rats, but m6A methylation enriched mRNA sequences at 3'UTR and 5'UTR. Paramasivan et al. (104) suggested that targeting m6A through METTL3 and FTO might be a potential diagnostic or therapeutic strategy for hypertension in the future. These results reinforce the potential role of m6A in blood pressure regulation, but the underlying mechanism is yet to be elucidated.

### 4.2 m6A in pulmonary hypertension

PH is characterized by remodeling of small pulmonary arteries and progressive increase in pulmonary vascular resistance, which ultimately leads to right heart failure and death. Research reveals that m6A methylation is significantly down-regulated in a rat model of PH; this may be related to the continuous low expression of METTL3, which affects the m6A level of PH-related genes such as Noggin (Nog), VANGL planar cell polarity protein 2 (Vangl2), and tricho-rhino-phalangeal syndrome 1 (Trps1) (105). In a monocrotaline-induced PH rat model, down-regulation of FTO expression and up-regulation of YTHDF1 expression were found to play a dominant role in mRNA m6A modification and participate in the pathogenesis of PH by regulating inflammation, glycolysis, TGF-β family receptor members, ECM receptor interactions, and platelet-derived growth factor (PDGF) signaling pathway (106). Zhou et al. (107) found that down-regulation of m6A modification through the down-regulation of METTL3 and METTL14 decelerated the progression of PH by inhibiting the proliferation and migration of pulmonary artery smooth muscle cells (PASMCs). Moreover, the level of the m6A-binding protein YTHDF2 was found to be significantly elevated in PASMCs under hypoxic conditions, and it recognized m6A-modified PTEN mRNA and promoted PTEN degradation, which in turn activated the PI3K/Akt signaling pathway, leading to hyperproliferation of PASMCs (108). In addition to mRNA, circRNAs also contain abundant m6A, and the abundance of m6A in circRNAs is significantly reduced in rats with hypoxia-induced PH. m6A methylation affects the progression of PH through circXpo6 and circTmtc3 in the circRNA-miRNA-mRNA network (109). Notably, m6A methylation is a biomarker for potential epigenetic modifications that regulate pulmonary arterial pressure; however, further studies are needed to determine the specific regulatory mechanisms by which m6A methylation is involved in PH and to facilitate the development of novel therapeutic approaches.

### 4.3 m6A in atherosclerosis

Atherosclerosis, which is the leading cause of cardiovascular deaths worldwide, is characterized by fibrous proliferation, chronic inflammation, lipid metabolism and accumulation, oxidative stress, and immune dysregulation. Emerging evidence suggests that atherosclerosis is also a disease with multiple epigenetic mechanisms.

In recent years, many studies have demonstrated that m6A methylation can regulation the progression of atherosclerosis through post-transcriptional modulation of RNAs (77, 110). Wu et al. (111) found a significant reduction in m6A methylation in peripheral blood leukocytes of patients and mice with atherosclerosis. Bioinformatics analysis also suggests that the pathogenesis of atherosclerosis involves differential expression of methylated genes. Previous studies have shown that METTL3-mediated methylation is involved in the inflammatory cascade response in endothelial cells (ECs) and is closely associated with hemodynamics and the pathogenesis of atherosclerosis (73). METTL3 is up-regulated during the proliferation and migration of human coronary smooth muscle cells (HCASMCs) and is involved in the pathogenesis of atherosclerosis by affecting protein synthesis and energy metabolism (112). In addition, Dong et al. (113) explored the role and molecular mechanism of m6A-METTL3 in a mouse model of atherosclerosis, which showed that the knockdown of METTL3 inhibited the development of endothelial atherosclerosis through the Janus Kinase 2/Signal Transducer and Activator of Transcription 3 (JAK2/STAT3) pathway and IGF2BP1, thus preventing the progression of atherosclerosis. Li et al. (114) reported that METTL3 promoted m6A-dependent degradation of epidermal growth factor receptor (EGFR) mRNA, thereby attenuating the progression of atherosclerosis. Furthermore, Tang et al. found that METTL3-mediated m6A modification enhanced lncRNA H19 stability, leading to endothelial cell inflammation and pyroptosis in atherosclerosis (115).

Similarly, METTL14 also plays a vital role in the pathogenesis of atherosclerosis. It was found that the knockdown of METTL14 significantly inhibited ECs and atherosclerotic plaque formation, suggesting the therapeutic potential of METTL14 in atherosclerosis (77). Furthermore, Zhang et al. (110) suggested that METTL14 increased the level of m6A methylation processing in pri-miR-19a, thereby promoting the proliferation and invasion of vascular ECs and the progression of atherosclerosis. Tang et al. (116) found that METTL14 affected the m6A methylation of lncRNA ZFAS1, which is involved in cholesterol metabolism and vascular inflammation, consequently affecting the development of atherosclerosis.

### 4.4 m6A in ischemia/reperfusion injury

Recovery of blood flow after myocardial ischemia causes damage to cardiomyocytes by inducing oxidative stress and releasing oxidative free radicals. As the most enriched RNA modification, m6A methylation is significantly up-regulated in hypoxia-induced cardiomyocyte injury. Song et al. (60) found that m6A modification and METTL3 expression were increased in cardiomyocytes under H/R conditions and cardiomyocytes in I/R-injured mice. Wang et al. (117) found that the level of METTL3 was up-regulated in I/R rats and cardiomyocytes; METTL3 exacerbated I/R injury in cardiomyocytes by mediating DiGeorge syndrome critical region 8 (DGCR8) to promote the expression of miR-143-3p in an m6A-dependent manner, which further reduced the expression of protein kinase C epsilon. Ye et al. (118) reported that m6A methylation of the 5'-UTR of hypoxia-associated mRNAs was up-regulated through hypoxia-induced overexpression of METTL3, which promoted the localization of eIF4A2 to target genes and facilitated the translation of these genes in hypoxic myocardium, thereby attenuating hypoxia-induced injury. In addition, a study showed that WTAP promoted endoplasmic reticulum stress and apoptosis in I/R-treated rat hearts, activated mRNA m6A of activating transcription factor 4 (ATF4), and up-regulated its expression, thereby exacerbating myocardial I/R damage (119). Pang et al. (120) found that METTL14 attenuated ischemia/reperfusion injury by regulating Wnt/β-Catenin signaling pathway, significantly improved myocardial function and reduced myocardial infarct size, serum lactate dehydrogenase (LDH) level and cardiomyocyte apoptosis.

### 4.5 m6A in myocardial infarction

MI refers to myocardial necrosis caused by acute or sustained ischemia and hypoxia in the coronary arteries. Cheng et al. (121) found that m6A modification was significantly reduced in the hearts of MI mice, with ALKBH5 being a critical regulator. Knockdown of ALKBH5 reduces the level of LDH and creatine kinase MB (CK-MB) and improves systolic and diastolic function in acute MI mice. Mathiyalagan et al. (122) found that the FTO level was down-regulated within 4 h after MI was induced in mice and that increased FTO expression helped to protect and restore cardiomyocyte function by improving cardiac homeostasis in mice with cardiac remodeling shortly after MI, with the effect persisting for nearly a week. Gong et al. (123) found that both knockdown and knockout of METTL3 enhanced cardiomyocyte proliferation, reduced cell size and improved cardiac function after MI. This might be related to the fact that silencing of METTL3 decreased m6A-mediated maturation of miR-143-3p, which increased the level of miR-143-3p targets, thereby promoting cardiomyocyte proliferation. Furthermore, FTO, YTHDF3 and ZC3H13 were also found differentially expressed in MI tissues; based on the different expression profiles of m6A regulators, molecular subtypes of MI with different clinical features were identified, which might provide new clues for exploring potential biomarkers of different types MI.

### 4.6 m6A in cardiac hypertrophy

Cardiac hypertrophy is usually characterized by enlarged cardiomyocytes and thickening of ventricular walls. Initially, this growth is an adaptive response to maintain cardiac function; however, under sustained stress, these changes become maladaptive over time, eventually leading to heart failure. In addition to common mechanisms such as calcium handling, autophagy, oxidative stress and inflammation, gene transcription is a new research hotspot in cardiac hypertrophy (124). A study by Dorn et al. (125) demonstrated that specific types of m6A modification in proteins were increased upon stimulation of cardiomyocytes using hypertrophic signals. METTL3-regulated methylation led to compensatory cardiac hypertrophy, which was critical for the pathological process of hypertrophy. Furthermore, METTL3 knockout was found to promote eccentric cardiomyocyte remodeling and dysfunction in mice. These findings suggest that METTL3-mediated dynamic methylation and modification are required to regulate cardiomyocyte growth responses (21). Xu et al. (126) found that the expression of YTHDF2 was increased in cardiac hypertrophy and that YTHDF2 inhibited cardiac hypertrophy by degrading the cardiac hypertrophy marker MYH7 mRNA via m6A-dependent mRNA. Qian et al. (127) found that IGF2BP2 recognizes the m6A modification in the target sequence of miR-133a and subsequently forms a complex with argonaute 2 (AGO2), which promotes the accumulation of miR-133a on the target mRNAs and thus prevents cardiac hypertrophy.

### 4.7 m6A in heart failure

HF is the leading cause of death in patients with end-stage heart disease and is characterized by decreased cardiac function, fatigue, and reduced exercise tolerance. The pathological process of HF includes altered levels of cardiomyocyte apoptosis, myocardial fibrosis, and altered gene expression. The levels of mRNA and protein expression in the right ventricle are inconsistent during HF, suggesting a role of post-transcriptional regulation in HF (128). Mathiyalagan et al. (122) found that FTO was vital in cardiac contractile function during homeostasis and remodeling. This study found increased m6A modification and significantly decreased FTO expression in infarct and peri-infarct regions of failing hearts, as compared with healthy heart tissues. FTO overexpression attenuated the ischemia-induced increase in m6A modification. A more rapid progression of HF was witnessed with a lower ejection fraction and more severe dilatation in FTO knockout mice, indicating the indispensable role of FTO in HF. Zhang et al. (129) showed that the level of FTO was significantly reduced in both HF mice and HF patients; however, the overexpression of FTO significantly attenuated cardiac dysfunction by modulating glycolysis and glucose uptake in HF mice. Differently expressed genes of m6A methylation are also involved in the development of HF. Hypermethylated and hypomethylated transcripts are linked to different biological processes. Gene ontology (GO) analysis has revealed that differentially m6A-modified transcripts are mainly involved in metabolism and cardiac signaling in HF. m6A-seq has revealed differentially methylated transcripts of epigenetic proteins, transcription factors, and upstream regulators of signaling pathways, indicating that m6A methylation may be involved in the regulation of gene expression in HF (130). These findings suggest abnormal m6A methylation in failing hearts, indicating that aberrant m6A methylation may serve as a potential biomarker and therapeutic target by modulating m6A modulators and downstream genes.

In summary, different m6A regulators have different biological effects in cardiovascular diseases. Even the same class of methylation transferase may have different biological effects in the same disease but with different molecular isoforms. This may be related to the fact that this rich modification involves the regulation of many different processes, but the identification of disease-specific deterministic modifications of m6A is probably what we should focus on next.

## 5 Targeting m6A modification with active natural products for the treatment of CVDs

**Figure 3:**
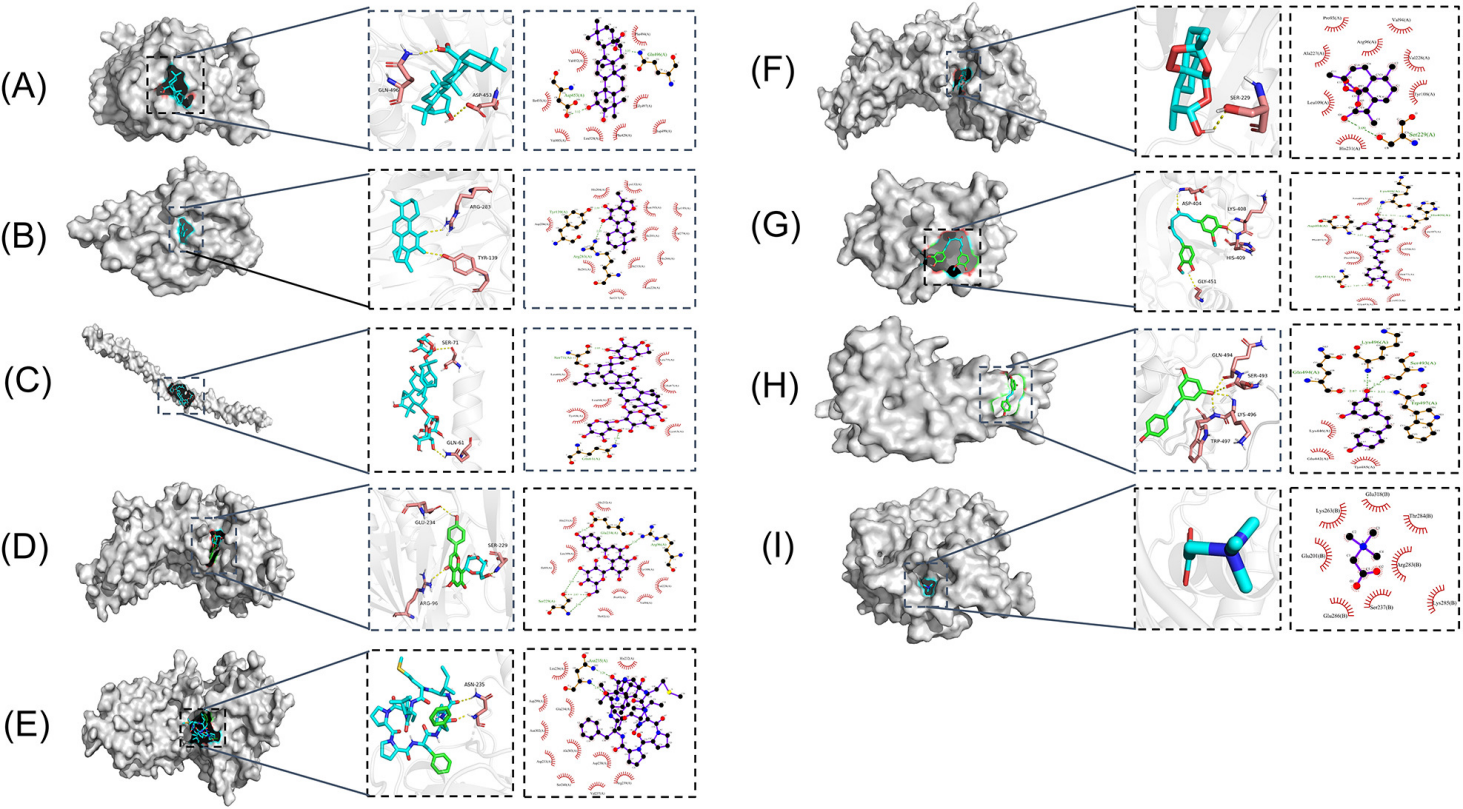
Molecular models of natural products binding to m6A regulators. **(A)** Maslinic Acid and METTL3. **(B)** Tanshinone IIA and ALKBH5. **(C)** Notoginsenoside R1 and WTAP. **(D)** Vitexin and FTO. **(E)** Cyclolinopeptide B and FTO. **(F)** Dihydroartemisinin and FTO. **(G)** Curcumin and YTHDF2. **(H)** Resveratrol and YTHDF3. **(I)** Betaine and METTL14.

Natural products can serve as chemical libraries for drug discovery and treating cardiovascular diseases. In this section, we will focus on the mechanisms of natural ingredients in traditional drugs targeting m6A regulators and their current role in the treatment of cardiovascular diseases (see details in Table 3 and Figure 3).

**Table 3:** The role of natural products targeting m6A modification in the treatment of cardiovascular diseases.

Natural product	Structure	m6A regulator(s)	Molecular mechanism(s)	Application(s)	Reference
Maslinic acid	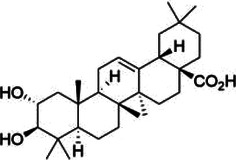	METTL3	Inhibits of cardiac hypertrophy	Cardiac hypertrophy	(134)
		ALKBH5	Inhibits the production of ROS and pro-inflammatory cytokines	Diabetes-associated atherosclerosis	(135)
Tanshinone IIA	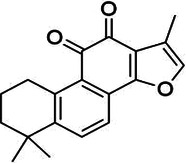	ALKBH5	Inhibits of modification of m6A of galectin-3	Cardiac hypertrophy	(145)
Total Panax notoginseng saponin	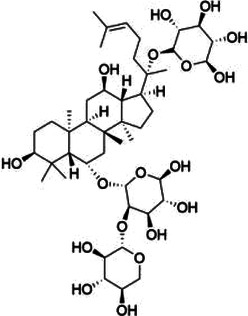	WTAP	Leads to vascular smooth muscle cell proliferation, migration and intimal hyperplasia.	Arterial restenosis	(152)
Vitexin	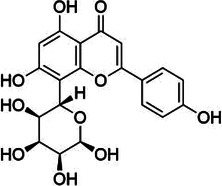	FTO	Down-regulates the expression of pro-inflammatory factors and proteins and increases the expression of the tight junction proteins	Vascular endothelial cell barrier injury	(155)
Methionine cyclic peptides	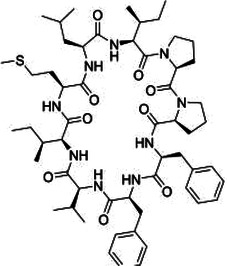	FTO	Inhibits the expression of inflammatory factors	Cardiovascular inflammation	(162)
Dihydroartemisinin	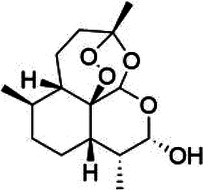	FTO	Inhibits AngII-induced cell proliferation and inflammation	Atherosclerosis	(168)
Curcumin	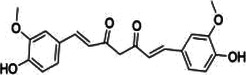	METTL3, METTL14, ALKBH5, FTO, and YTHDF2	Exerts a protective role against lipopolysaccharide-induced lipid metabolism disorder in piglets	Lipid metabolism disorder	(174)
Resveratrol	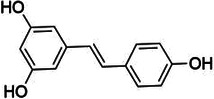	YTHDF2, YTHDF3	Regulates of transcriptional PPARα activity	Lipid metabolism disorder	(92)
Betaine	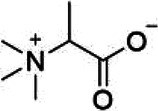	FTO	Targeting PGC1α and reducing its m6A level on CDS transcripts	Lipid deposition	(188)
		METTL14	Attenuates EOM-induced ROS overproduction, mitochondrial damage, apoptosis and cardiac defects	Cardiac defects	(189)

### 5.1 Maslinic acid

Maslinic acid (MA), a pentacyclic triterpenoid rich in olive pericarp with a wide range of pharmacological properties, has been shown to inhibit inflammatory responses with strong anti-tumor, anti-bacterial, and antioxidant effects (131). Studies have demonstrated the potential of MA in blood glucose reduction, induction of apoptosis of human cancer cells, and protection against CVDs (132, 133). MA has been found to significantly inhibit cardiac hypertrophy both *in vitro* and *in vivo*. *in vitro*, MA significantly inhibits Ang II-induced hypertrophy of neonatal murine cardiomyocytes; *in vivo*, MA can significantly improve cardiac function and reduce cardiomyocyte area and expression of hypertrophic markers in a mouse model of cardiac hypertrophy. The study further confirmed the critical role of METTL3-mediated m6A methylation in the inhibition of cardiac hypertrophy by MA, as overexpression of METTL3 could reverse the inhibitory effect of MA on cardiac hypertrophy (134). MA also promoted the recruitment of the demethylase ALKBH5 to thioredoxin-interacting protein (TXNIP) mRNA and subsequently enhanced its m6A demethylation, reduced the stability of TXNIP mRNA, and inhibited the production of ROS and pro-inflammatory cytokines, which alleviated high glucose-induced endothelial cell inflammation and injury. This may provide new insights into the potential therapeutic effect of MA in the prevention of inflammatory diseases such as diabetes-associated atherosclerosis (135).

### 5.2 Tanshinone IIA

Tanshinone IIA is an active ingredient extracted from *Salvia miltiorrhiza*. It is a diterpene quinone, a component of various biochemical reactions and biological activities in the plant (136–139). Tanshinone IIA has a wide range of clinical applications, and previous studies have demonstrated that Tanshinone IIA helps to prevent platelet aggregation, free radical damage, and cardiac arrhythmias, lower blood viscosity, and improve microcirculation (140–143). Tanshinone IIA modulates vascular macrophages to ameliorate atherosclerosis, enhances the expression of signals related to cytosolic burial, and promotes macrophage clearance of apoptotic cells (144). Zhang et al. found that Tanshinone IIA significantly inhibited Ang II-induced hypertrophy *in vitro* and TAC-induced cardiac hypertrophy *in vivo*; this mechanism was associated with Tanshinone IIA inhibition of ALKBH5-mediated m6A modification of galectin-3 (145). Interestingly, Tanshinone IIA injection has been widely used to treat CVDs in China. Several meta-analyses and systematic evaluations have shown that Tanshinone IIA injection, in combination with conventional medications, can significantly reduce myocardial oxygen consumption and ventricular remodeling and further improve the prognosis of patients (146–149). However, its association with methylation modification still needs to be further explored.

### 5.3 Total Panax notoginseng saponin

Total Panax notoginseng saponin (TPNS) is the main active ingredient of *Panax notoginseng* (Burk) E, H, Chen (Araliaceae) with a long history of use in the clinical treatment of cardiovascular diseases; it has also been widely applied and studied for its antithrombotic, anti-atherosclerotic, anti-inflammatory, antioxidant, and endothelial cell-protective properties (150). By prophylactically injecting a high dose of Panax ginseng saponin into mice with endotoxin LPS-induced myocardial injury, a study found that pretreatment with TPNS could significantly reduce the number of inflammatory cells, such as neutrophils and macrophages, and inhibit the expression of inflammatory factors, which could effectively ameliorate myocardial damage (151). Studies have shown that TPNS inhibits the intimal hyperplasia and reverses the reduced m6A quantity in balloon catheter-injured rat carotid artery. TPNS also up-regulates WTAP expression and regulates the WTAP/p16 signals via m6A, leading to the proliferation and migration of vascular smooth muscle cells as well as intimal hyperplasia, thus suggesting a new theoretical basis for application in arterial restenosis (152).

### 5.4 Vitexin

Vitexin is a natural free radical scavenger with the ability to provide electrons; it is a C-glycoside flavonoid that can be found in ouabain, hawthorn, passion flower, sandalwood, echinocereus, and mung bean, and is the main flavonoid found in food sources. Dong et al. (153) found that pretreatment of cultured neonatal rat cardiomyocytes with vitexin before hypoxia and reoxygenation could increase cell viability and extracellular signal-regulated protein kinase activity by reducing apoptotic cells and intracellular Ca^2+^ overload. Dong et al. (154) also found that vitexin had a cardioprotective effect in I/R rats, as vitexin-treated rats had reduced ST-segment elevation on electrocardiography and decreased area of myocardial infarction. The damage of vascular endothelial cell barrier induced by chronic low-grade inflammation is a major cause of CVDs. Gan et al. (155) found that vitexin could combat low-level inflammation in vascular endothelial cells and improve vascular endothelial barrier function. The specific molecular mechanism of vitexin involves direct binding to FTO, which reduces the level of m6A methylation modification. This reduction down-regulates the expression of pro-inflammatory factors and proteins, and concurrently increases the expression of tight junction proteins such as claudin-1 (CLDN1), occludin, and Zonula Occludens-1 (ZO-1), thereby improving the vascular endothelial cell barrier function.

### 5.5 Methionine cyclic peptides

Methionine cyclic peptides (MCP) are a class of cyclic hydrophobic peptides derived from flaxseed, an important oilseed crop that has a long history of cultivation and is a high-quality source of plant-based *ω*-3 fatty acids. Studies have shown that supplementation with flaxseed oil reduces serum triacylglycerol and cholesterol, thereby reducing the risk of CVDs (156, 157). Parikh et al. found that the addition of flaxseed to a high-lipid and high-glucose diet significantly reduced myocardial ischemic lesions, decreased the frequency of ischemic lesions in rats with hereditary obesity and the metabolic syndrome, reduced cholesterol levels, and improved cardiac systolic and diastolic function (158). MCP has excellent antioxidant, antihypertensive, anti-inflammatory, and immunosuppressive effects (159–161). Studies have shown that high-purity MCP obtained using silica gel chromatography combined with liquid chromatography separation technique can effectively inhibit the expression of inflammatory factors in trimethylamine N-oxide (TMAO)-induced human umbilical vein endothelial cells and reduce the level of inflammatory factors and m6A content in live mouse model, thus exerting anti-cardiovascular inflammation effects. Moreover, an inhibitor of FTO, entacapone, was found to attenuate the anti-cardiovascular inflammation effect of MCP, implying that the anti-inflammatory effect of MCP may be achieved through significant activation of the FTO-mediated m6A demethylation pathway (162).

### 5.6 Dihydroartemisinin

Dihydroartemisinin (DHA), a semi-synthetic derivative of artemisinin, is a more water-soluble and potent anti-malarial drug than artemisinin. DHA inhibits the proliferation, migration, inflammation, and tube formation of cultured endothelial cells (163–165). The antiproliferative effect of DHA may be attributed to its association with the vascular endothelial growth factor (VEGF) signaling pathway in its interaction (166). DHA reduces the proliferation and inflammation of high-glucose induced vascular smooth muscle cells by inhibiting the miR-376b-3p/KLF15 pathway (167). DHA has also shown to inhibit Ang II-induced cell proliferation and inflammation and suppress FTO expression promoted by Ang II in a dose-dependent manner. This potential mechanism may be related to the down-regulation of FTO by DHA, which promotes the expression of nuclear receptor subfamily 4 group A member 3 (NR4A3) via mRNA demethylation. This may provide a new insight into the mechanisms of DHA and its critical role in the pathogenesis of atherosclerosis (168).

### 5.7 Curcumin

Curcumin, a polyphenol from the rhizome of turmeric, has attracted increasing attention in the medical field for its potential antioxidant, anti-inflammatory, and anti-cancer properties. A growing body of evidence suggests that curcumin prevents and mitigates a variety of chronic diseases such as neurodegenerative, inflammatory, and cardiovascular diseases (169, 170). A study found that dietary supplementation with curcumin reduced fasting blood glucose and improved insulin sensitivity in obese rats (171). Curcumin inhibits adipogenesis by blocking the mitotic clonal expansion process and up-regulating adipocyte energy metabolism and apoptosis (172, 173). Curcumin also suppressed the LPS-induced elevation of Sterol Regulatory Element Binding Protein-1C (SREBP-1c) and Stearoyl-CoA Desaturase-1 (SCD-1) mRNA levels, affected the expression of METTL3, METTL14, ALKBH5, FTO, and YTHDF2 mRNAs, and increased the abundance of m6A in LPS-treated liver, thereby exerting a protective effect against LPS-induced lipid metabolism disorder in piglets (174). Curcumin was also found to significantly inhibit ROS production and apoptosis through the inhibition of I/R and improve cell viability by regulating the expression of m6A-associated proteins and decreasing the total m6A level in cardiomyocytes (175, 176).

### 5.8 Resveratrol

Resveratrol, a natural polyphenolic compound found in plants, has been found to demonstrate antioxidative, anti-inflammatory, anticarcinogenic, and antibacterial effects (177–179). Accumulating evidence suggests that resveratrol helps to attenuate abnormal lipid metabolism. Ran et al. (180) found that the regulatory role of resveratrol in the lipid metabolism balance of zebrafish under dietary stress conditions was associated with the AMP-activated protein kinase α (AMPKα) pathway. Resveratrol also improves serum lipid characters and reverses body fat deposition in a pig model (181). In a study by Sun et al. (182), resveratrol was found to restore clock-mediated dysfunctional lipid metabolism in high-fat-fed mice through the activation of clock machinery. A recent study has demonstrated that resveratrol intake beneficially affects the transcriptional level of lipid metabolic genes, especially PPARα, and alleviates hepatic lipid accumulation as well as elevation of mRNA levels of m6A methylases and demethylases, increase of YTHDF2 expression, and obvious reduction of YTHDF3 mRNA expression and m6A level. Thus, the protective role of resveratrol in maintaining lipid metabolism may be attributed to the regulation of transcriptional PPARα activity and the modification of m6A methylated lipid metabolism-related genes (92). However, further investigation is warranted to explore the precise crosstalk between resveratrol-regulated lipid homeostasis and m6A RNA methylation.

### 5.9 Betaine

Betaine is a water-soluble quaternary amine-type alkaloid found in various foods including wheat germ, beet, spinach, and wolfberry; it plays a significant role as an important methyl donor and osmotic pressure regulator in several physiological activities in the mammalian body (183). Numerous studies have shown that betaine has anti-inflammatory effects, increases free fat oxidation, anti-lipogenesis, mitochondrial function, and improves insulin resistance (184–186). Since betaine is a methyl donor, some researchers have proposed that epigenetic modification regulation may be a mechanism through which betaine exerts its pharmacological effects. Studies have shown that betaine supplementation during adolescence can protect female mice from high blood lipids through FTO-dependent m6A demethylation (187). Betaine-induced FTO overexpression promotes mitochondrial biogenesis and further improves lipid deposition by targeting PPARγ Coactivator 1-α (PGC1-α), reducing its m6A level on CDS transcripts (188). Another study found that extractable organic matter (EOM) extracted from particulate matter 2.5 (PM2.5) significantly reduced the overall m6A RNA methylation level in the hearts of zebrafish larvae through aryl hydrocarbon receptor (AHR)-mediated downregulation of METTL14, thereby upregulating traf4a and bbc3 expression; the methylation levels could be restored by the methyl donor betaine. EOM-induced ROS overproduction, mitochondrial damage, apoptosis, and cardiac defects were also found to be attenuated by the methyl donor betaine (189).

## 6 Conclusions and future perspectives

m6A is an important epigenetic modification of RNA that has garnering increasing attention in the study of CVDs in recent years. This review focused on the relationship between various CVDs and m6A and its related regulators, suggesting that the level of m6A-modified RNA is regulated by methyltransferases and demethyltransferases, and that m6A recognition proteins can regulate the splicing, translation, and degradation of m6A-modified RNA, thus affecting the expression of corresponding proteins. Among them, METTL3, METTL14, WTAP, YTHDF1–3, YTHDC1, FTO, and ALKBH5 are the main regulators involved in m6A modification. m6A elicits a wide range of effects on RNAs through changes in RNA stability, conformation and folding, or direct regulation of the interactions between modified RNAs and binding proteins that affect RNA fate and function. Changes in m6A-related genes or proteins affect a variety of biological processes involving m6A methylation in CVDs, including apoptosis, autophagy, pyroptosis, and ferroptosis. However, the mechanisms of m6A affecting these biological processes are complex and currently unclear. For example, ALKBH5 positively regulates apoptosis, whereas FTO does not. Similar differences were found regarding pyroptosis, with METTL3 enhancing polarization and immune function while METTL14 suppressing these processes. Given so many different functions of m6A, further studies in this field may focus on distinguishing between the direct consequences of such modifications and the secondary or tertiary consequences that have been inappropriately attributed to m6A. Therefore, independent validation of the function of m6A is necessary and will play an essential role in improving our overall understanding of the biologically important aspects of m6A. With the advancement of research on methylation-specific epigenetic modifications, precision medicine by m6A modification-targeted epigenetic editing, may pose new solutions for CVDs.

Considering the wide range of promising applications of natural products in the research and treatment of diseases, a large number of natural products have been studied and trialed in the clinical treatment and food therapies for CVDs. However, relatively few studies have been conducted on the regulation of m6A modification by natural products, and the roles and mechanisms of their epigenetic modifications in CVDs remain to be further explored. Moreover, using advanced technologies such as artificial intelligence to predict the interactions between natural products and m6A regulators, and to elucidate their potential binding affinities and regulatory mechanisms, could provide more accurate candidates for experimental research. In addition, most of existing studies are based on animal experiments, resulting in a lack of validation in large-scale, long-term clinical studies. These limitations emphasize the need for further research to bridge the gap between basic medical experiments and clinical translation. Hopefully, researchers will further explore and elucidate the relationship between methylation-related processes and CVDs, and investigate the use of natural plant compounds in the prevention and treatment of CVDs.

## Glossary

CVDs: cardiovascular diseases
PH: pulmonary hypertension
MI: myocardial infarction
HF: heart failure
PCI: percutaneous coronary intervention
mRNA: messenger RNA
ncRNAs: non-coding RNA
m6A: N6-methyladenosine
m1A: N1-methyladenosine
m5C: 5-methylcytosine
rRNAs: ribosomal RNAs
tRNAs: transfer RNAs
miRNAs: micro RNAs
lncRNAs: long non-coding RNAs
3' UTRs: 3'-untranslated regions
5' UTRs: 5'-untranslated regions
MTC: methyltransferase complex
METTL3: methyltransferase-like3
METTL14: methyltransferase-like14
WTAP: Wilms tumor 1 associated protein
ZC3H13: zinc finger CCCH-type containing 13
VIRMA: vir like m6A methyltransferase associated
RBM15: RNA-binding motif protein 15
FTO: fat mass and obesity-associated
ALKBH5: alpha-ketoglutarate-dependent dioxygenase alkB homolog 5
ALKBH3: alpha-ketoglutarate-dependent dioxygenase alkB homolog 3
RBPs: m6A RNA-binding proteins
YTHDF1/2/3: YTH domain-containing family protein 1–3
YTHDC1/2: YTH domain-containing protein 1–2
eIF3: eukaryotic initiation factor 3
HNRNP: heterologous nuclear ribonucleoprotein
IGF2BPs: insulin-like growth factor 2 mRNA binding proteins
MeRIP-Seq: methylated RNA immunoprecipitation and next-generation sequencing
GLORI: glyoxal and nitrite-mediated deamination of unmethylated adenosine
I/R: ischemia/reperfusion
TFEB: transcription factor EB
MHTR: myosin heavy chain-associated RNA
FOXO3: post-transcriptional forkhead box O3
ULK1: Unc-51-like autophagy-activated kinase 1
mTOR: mammalian target of the rapamycin
LC3BII: light chain 3B II
H/R: hypoxia/reoxygenation
PTEN: phosphatase and tensin homolog
PI3K/Akt/GSK-3β: phosphatidylinositol 3-kinase/protein kinase B/glycogen synthase kinase-3
NLRP3: NOD-like receptor protein 3
ASC: apoptotic-associated speck-like protein containing a CARD
ACS: acute coronary syndrome
IRF-1: IFN regulatory factor-1
DGCR8: DiGeorge syndrome critical region 8
NEAT1: nuclear paraspeckle assembly transcript 1
TINCR: terminal differentiation-induced non-coding RNA
NF-κB: nuclear transcription factor κB
LPS: lipopolysaccharide
FOXO1: forkhead box O1
IL-6: interleukin-6
TNF-α: tumor necrosis factor α
IL-1β: interleukin-β
STAT1: signal transducer and activator of transcription 1
PPAR-γ: peroxisome proliferator-activated receptor
Hmox1: heme oxygenase 1
ROS: reactive oxygen species
MG53: mitsugumin 53
FSS: fluid shear stress
ERRβ: estrogen receptor related β
NFAT5: nuclear factor of activated T cells 5
HIF1α: hypoxia-inducible factor 1-α
NFE2L2: nuclear factor erythroid 2-related factor 2
SR-B1: scavenger receptor B-type 1
KCNQ1OT1: KCNQ1 opposite strand/antisense transcript 1
BACH1: BTB domain and CNC homolog 1
HMEC-1: human microvascular endothelial cell line-1
ACSL4: acyl-CoA synthetase long-chain family member 4
GSH: glutathione
GPX4: glutathione peroxidase 4
Nog: noggin
Vangl2: VANGL planar cell polarity protein 2
Trps1: tricho-rhino-phalangeal syndrome 1
PDGF: platelet-derived growth factor
PASMCs: pulmonary artery smooth muscle cells
ECs: endothelial cells
JAK2/STAT3: Janus Kinase 2/Signal Transducer and Activator of Transcription 3
EGFR: epidermal growth factor receptor
ATF4: activating transcription factor 4
LDH: lactate dehydrogenase
CK-MB: creatine kinase MB
AGO2: argonaute 2
GO: gene ontology
MA: maslinic acid
TXNIP: thioredoxin-interacting protein
TPNS: total panax notoginseng saponin
CLDN1: claudin-1
ZO-1: zonula occludens-1
MCP: methionine cyclic peptides
TMAO: trimethylamine N-oxide
DHA: dihydroartemisinin
VEGF: vascular endothelial growth factor
NR4A3: nuclear receptor subfamily 4 group A member 3
SREBP-1c: sterol regulatory element binding protein-1C
SCD-1: stearoyl-CoA desaturase-1
AMPKα: AMP-activated protein kinase α
PGC1-α: PPARγ coactivator 1-α
PM2.5: particulate matter 2.5
AHR: aryl hydrocarbon receptor
